# Beyond the Eye: Glaucoma and the Brain

**DOI:** 10.3390/brainsci15090934

**Published:** 2025-08-28

**Authors:** Marco Zeppieri, Federico Visalli, Mutali Musa, Alessandro Avitabile, Rosa Giglio, Daniele Tognetto, Caterina Gagliano, Fabiana D’Esposito, Francesco Cappellani

**Affiliations:** 1 Department of Ophthalmology, University Hospital of Udine, 33100 Udine, Italy; 2 Department of Medicine, Surgery and Health Sciences, University of Trieste, 34129 Trieste, Italy; 3 Department of Ophthalmology, University of Catania, 95123 Catania, Italy; 4 Department of Optometry, University of Benin, Benin 300283, Nigeria; 5 Department of Ophthalmology, Africa Eye Laser Center Ltd., Benin 300211, Nigeria; 6 Faculty of Medicine, University of Catania, 95123 Catania, Italy; 7 Department of Medicine and Surgery, University of Enna “Kore”, 94100 Enna, Italyfrancesco.cappellani@unikore.it (F.C.); 8 Eye Center “G.B. Morgagni-DSV”, 95100 Catania, Italy; 9 Imperial College Ophthalmic Research Group (ICORG) Unit, Imperial College, 153-173 Marylebone Rd., London NW1 5QH, UK

**Keywords:** glaucoma, neurodegeneration, retinal ganglion cells, central nervous system, neuroprotection

## Abstract

Glaucoma is traditionally classified as an ocular disease characterized by progressive retinal ganglion cell (RGC) loss and optic nerve damage. However, emerging evidence suggests that its pathophysiology may extend beyond the eye, involving trans-synaptic neurodegeneration along the visual pathway and structural changes within central brain regions, including the lateral geniculate nucleus and visual cortex. In this narrative review, we have used the phrase ‘brain involvement’ to underscore central changes that accompany or follow retinal ganglion cell loss; we have not intended to redefine glaucoma as a primary cerebral disorder. Neuroimaging studies and neurocognitive assessments in adult glaucoma patients, primarily older individuals with primary open-angle glaucoma reveal that glaucoma patients may exhibit alterations in brain connectivity and cortical thinning, aligning it more closely with neurodegenerative disorders such as Alzheimer’s and Parkinson’s disease. This evolving neurocentric perspective raises important questions regarding shared mechanisms—such as mitochondrial dysfunction, chronic inflammation, and impaired axonal transport—that may link glaucomatous optic neuropathy to central nervous system (CNS) pathology. These insights open promising therapeutic avenues, including the repurposing of neuroprotective and neuroregenerative agents, targeting not only intraocular pressure (IOP) but also broader CNS pathways. Furthermore, neuroimaging biomarkers and brain-targeted interventions may play a future role in diagnosis, prognosis, and individualized treatment. This review synthesizes current evidence supporting glaucoma as a CNS disease, explores the mechanistic overlap with neurodegeneration, and discusses the potential clinical implications of glaucoma within a neuro-ophthalmologic paradigm.

## 1. Introduction

Glaucoma has traditionally been viewed as an exclusively ocular condition marked by the progressive degeneration of retinal ganglion cells (RGCs) and optic nerve impairment, resulting in visual field defects. A growing body of evidence supports a more expansive neurodegenerative framework wherein glaucomatous injury transcends the ocular region and affects core brain systems. Neurodegenerative processes, including neuronal loss and white matter alterations, have been shown to occur throughout the visual pathway in glaucoma and may begin early in the disease course, potentially preceding or paralleling clinically detectable structural and functional ocular changes [1,2]. This paradigm shift challenges traditional clinical views and fosters the development of potential innovative diagnostic and treatment approaches aimed at both the brain and the eye. Consistent with contemporary clinical practice, glaucoma is classified as an optic neuropathy at the ocular level.

Structural brain imaging studies provide evidence for central nervous system involvement in glaucoma. Researchers have documented diminished gray matter volume and cortical thickness in the visual cortex and related areas by surface-based analysis (SBA) and voxel-based morphometry (VBM) [3]. These modifications are associated with the severity of visual field defects, RNFL thickness and the cup-to-disk ratio, suggesting that anatomical changes in the brain are not only incidental observations but may possess direct therapeutic significance [4]. Moreover, microstructural alterations in subcortical relay regions and optic radiations, as demonstrated by diffusion-tensor imaging, reinforce the hypothesis that glaucomatous degeneration advances trans-synaptically via the retino-geniculo-cortical pathway [5].

Functional imaging and sophisticated diffusion MRI investigations validate these anatomical observations by revealing impaired connections within the visual system. Diffusion tensor imaging (DTI) demonstrates reduced fractional anisotropy and increased mean diffusivity—indicators of white matter microstructural impairment—in the optic radiations and optic nerves [6,7]. Resting-state and task-based functional MRI demonstrate diminished activity and connectivity between primary and higher visual areas [8,9]. These functional abnormalities resemble those observed in other neurodegenerative disorders, including Alzheimer’s disease, Parkinson’s disease, Huntington’s disease, and amyotrophic lateral sclerosis, all of which are marked by progressive neuronal degeneration and network disintegration, supporting the concept that glaucoma could be considered as a neuro-ophthalmological syndrome encompassing both ocular and cerebral pathology.

The clinical ramifications of this broadened perspective are significant. Reconceptualizing glaucoma as a cerebral and ocular disorder allows for the identification of novel biomarkers through neuroimaging, the development of neuroprotective and neuroregenerative medicines aimed at central pathways, and the customization of management to encompass both ocular and cerebral health. The following sections of this review will investigate the mechanisms underlying brain involvement, assess potential overlaps with other neurodegenerative diseases, address methodological challenges and future directions.

## 2. Methods

This review employs a structured narrative method to synthesize existing knowledge about the role of central nervous system structures in glaucoma. A thorough literature search was performed in PubMed, Embase, Web of Science, and Scopus to identify relevant peer-reviewed articles published in English between January 2000 and June 2025. Search queries combined disease- and imaging-related terms using Boolean operators, for example: (“glaucoma” OR “glaucomatous optic neuropathy”) AND (“brain” OR “neurodegeneration” OR “cortical thinning” OR “visual pathway”) AND (“functional MRI” OR “diffusion tensor imaging” OR “magnetic resonance spectroscopy”). The objective was to obtain research that investigated the correlation between glaucoma and structural, functional, or metabolic alterations in the brain, specifically concerning regions associated with visual processing.

The eligibility criteria were meticulously established before the selection of the study. Included studies were required to assess brain anatomy or function in patients diagnosed with any subtype of glaucoma utilizing recognized neuroimaging methodologies. Research utilizing voxel-based morphometry, surface-based morphometry, diffusion tensor imaging, resting-state or task-based functional MRI, or magnetic resonance spectroscopy was prioritized. Only studies that had explicit methodological descriptions, and reported outcomes pertaining to alterations in gray matter, white matter, functional connectivity, or metabolic markers were included. Studies that just examined ophthalmic disease or treatments, without concurrent evaluations of the central nervous system, were excluded.

Papers deficient in imaging methods, clinical factors, or statistical analysis were excluded to ensure data quality. Duplicate publications and those with overlapping populations were detected and eliminated during the screening process.

Upon identifying the qualifying publications, data were retrieved regarding imaging modality, sample size, patient demographics, studied brain areas, and findings related to structural, functional, or metabolic problems. Instead of pursuing quantitative synthesis, the gathered information was categorized into specific domains: trans-synaptic degeneration, anatomical brain abnormalities, functional connectivity problems, metabolic results, and common pathways with other neurodegenerative illnesses. This thematic approach enabled a comprehensive analysis of the information, facilitating significant comparisons across research and contributing to the establishment of a coherent framework for understanding glaucoma as a condition impacting both the eye and the brain. Rather than delivering a comprehensive systematic analysis, this review presents an academic perspective informed by a synthesis of reliable data and practical clinical considerations.

## 3. Indications of Trans-Synaptic Degeneration

Recent neuroimaging and neuropathological studies have provided compelling evidence for trans-synaptic degeneration in glaucoma. This process, characterized by the transmission of neuronal injury from one neural structure to interconnected downstream regions, has been documented in the visual pathway of glaucoma patients. Retinal ganglion cells experience apoptosis due to increased intraocular pressure or other stresses, leading to cellular degeneration that propagates up the optic nerve, impacting the LGN and ultimately the primary visual cortex. Histological examinations have validated atrophy and neuronal degeneration in these areas, reinforcing the notion that glaucomatous damage propagates centrally via trans-neuronal processes [10,11].

Advanced neuroimaging methodologies have further substantiated this process. Research employing high-resolution structural MRI has demonstrated reduced LGN volume in glaucoma patients, frequently associated with disease severity and retinal nerve fiber layer thinning [12]. Concurrently, DTI has revealed impaired integrity in white matter pathways, including the optic radiation and optic tract, evidenced by reduced fractional anisotropy and elevated mean diffusivity, indicative of axonal degeneration and demyelination [13,14].

The subsequent impacts of this degeneration are also seen in cortical structures. Decreases in gray matter volume and cortical thickness have been noted in the primary visual cortex [15]. Functional deficits in these domains, evaluated using resting-state functional MRI, exhibit decreased activation and impaired connection with advanced visual processing centers. These modifications not only validate the dissemination of degeneration along anatomical pathways but also offer a physiological counterpart to structural impairment.

These data collectively suggest that trans-synaptic degeneration is an important pathogenic feature of glaucoma, linking ocular injury to widespread cerebral alterations. This process elucidates the multifocal characteristics of visual field loss and the engagement of brain regions beyond the traditional retino-geniculo-cortical circuit. It further underscores that a comprehensive understanding—and ultimately more effective treatment—of glaucoma will likely require consideration of its central nervous system component.

It is important to note several factors about the eye–brain continuum and differential concerns. The retina is a central nervous system tissue safeguarded by a blood–retinal barrier analogous to the blood–brain barrier. Damage at any point in the visual pathway can cause anterograde and retrograde trans-synaptic degeneration; however, this phenomena does not imply different underlying causes. Cerebral diseases, such as ischemic stroke, neurodegenerative disorders like Alzheimer’s disease, or brain tumors, can damage the optic radiations and visual cortex, resulting in homonymous field loss and subsequent retinal alterations without indicating glaucoma. Conversely, glaucomatous damage occurs in the anterior visual pathway and may progressively involve central changes. Understanding this distinction is crucial for clinical interpretation and for preventing nosological misinterpretation.

## 4. Morphological Modifications of the Brain

Structural neuroimaging has demonstrated evidence of structural alterations in the brain of individuals with glaucoma, surpassing traditional optic pathway deterioration. Cortical thinning in visual processing regions, especially in the occipital lobe, has been repeatedly recorded utilizing VBM and surface-based morphometry (SBM) methodologies [9,16]. The decreases in gray matter volume are associated with clinical indicators, indicating a connection between ocular pathology and cortical neurodegeneration [17]. Significantly, these alterations extend beyond the primary visual cortex to encompass association visual areas involved in advanced visual processing.

Furthermore, magnetic resonance imaging studies reveal that the LGN, a principal relay center between the retina and visual cortex, has significant atrophy in glaucoma patients relative to controls, even in the early stages of the disease [18]. The findings corroborate the trans-synaptic characteristics of glaucomatous neurodegeneration, since synaptic targets exhibit volume reduction subsequent to upstream retinal injury [19]. The optic tracts and optic radiations exhibit volume loss, corroborating diffusion metric studies and highlighting the extensive nature of structural involvement [20].

The structural modifications extend beyond primary visual pathways into intermediate and tertiary cerebral regions. The visual association cortices in the parietal and temporal lobes, which are crucial to sophisticated visual perception and spatial cognition, have reduced gray matter volume correlated with the severity of glaucoma [21]. This indicates that glaucomatous damage may compromise not just fundamental visual acuity but also advanced cognitive abilities including visual processing, spatial orientation, and attention. This extensive structural remodeling suggests that glaucoma is associated with larger cerebral alterations that may contribute to subtle abnormalities frequently seen in patients, such as delayed reaction times and challenges with visual-spatial activities.

Finally, surface-based investigations have revealed regional cortical thinning patterns that correspond to the distribution of glaucomatous damage [4,22]. Concurrent cortical thinning in both dorsal and ventral visual streams indicates that the “where” (spatial) and “what” (object recognition) processing pathways are vulnerable [23].

## 5. Alterations in Functional Connectivity

Besides structural degradation, glaucoma is now acknowledged to cause substantial disturbances in the brain’s functioning architecture. Resting-state functional magnetic resonance imaging (rs-fMRI) has proven pivotal in detecting connectivity problems within and outside the visual system. Numerous investigations have established functional decoupling between the primary visual cortex and higher-order association cortices. These changes are not simply secondary effects of diminished visual input but indicate fundamental dysregulation of neural networks. In early-stage glaucoma, diminished synchronization among visual networks can be observed, indicating that connection deficits may occur prior to or concurrently with structural alterations [8,24].

Furthermore, glaucoma patients demonstrate modified connections in non-visual areas, reinforcing the notion that the condition impacts wider brain systems. Disrupted integration has been noted between visual areas and regions associated with attention, spatial orientation, and cognitive control, such as the parietal cortex [25]. These findings align with documented deficits in attention, executive function, and reaction times noted in clinical evaluations of glaucoma patients. The non-visual connection alterations highlight glaucoma’s capacity to induce widespread neurological impairment, hence supporting its classification as a neurodegenerative condition.

Network-level investigations have elucidated the reconfiguration of cerebral function in glaucoma. Functional connection density mapping and graph theoretical methods reveal diminished global efficiency and compromised modular organization in visual and multisensory integration networks [26]. Patients demonstrate modified hub areas and diminished small-worldness, signifying a disruption in the conventional equilibrium between local specialization and global integration. The decline in functional efficiency may lead to a progressive deterioration in fundamental visual perception and complex activities, reflecting patterns seen in Alzheimer’s disease and other types of cortical neurodegeneration [27].

Some investigations indicate compensating enhancements in connection within intact regions, potentially signifying neuroplasticity or adaptive rearrangement. Increased connection within the default mode network (DMN) or between contralateral visual cortices has been discovered, presumably facilitating residual function in the early stages of the disease [17,28]. Nonetheless, the question of whether these modifications are advantageous or maladaptive continues to be a subject of controversy. Longitudinal investigations are essential to ascertain whether these compensatory responses can maintain visual performance or if they indicate network fatigue over time. Nevertheless, the existence of modified connection patterns in glaucoma underscores its categorization as a condition involving the central nervous system.

## 6. Metabolic and Neurochemical Observations

Recent studies on the metabolic and neurochemical alterations in glaucoma have yielded further evidence of central nervous system involvement. Magnetic resonance spectroscopy (MRS), a non-invasive imaging modality that detects biochemical changes in vivo, has revealed diminished levels of N-acetyl aspartate (NAA) in the visual cortex of individuals with glaucoma [29]. NAA serves as an indicator of neuronal vitality, and its decrease is seen as indicative of neuronal death or malfunction. These metabolic anomalies manifest in both early and severe stages of the disease and correspond with structural alterations and visual field metrics, suggesting that biochemical dysfunction accompanies or precedes morphological damage. Variations in myo-inositol concentrations have been seen as markers of glial activation, a phenomenon commonly observed in other neurodegenerative conditions such multiple sclerosis and Alzheimer’s disease. Moreover, the ratio of NAA to creatine, a dependable indicator of neurodegeneration, has been documented to decrease in visual and associative cortices in glaucoma, hence strengthening the disease’s neurochemical signature in the brain [30].

Additional metabolites, including choline and creatine, have been assessed in relation to glaucoma. Increased choline levels, frequently linked to heightened membrane turnover and gliosis, indicate reactive alterations in the brain following damage [31].

Mitochondrial dysfunction is acknowledged as a fundamental element of glaucomatous pathogenesis. Impaired mitochondrial oxidative phosphorylation, diminished ATP synthesis, and heightened formation of reactive oxygen species have all been associated with the degeneration of retinal ganglion cells and may also affect central neurons in the visual pathway [32].

Besides metabolic alterations, neuroinflammatory mechanisms are thought to play a role in the neurodegenerative cascade. Increased concentrations of pro-inflammatory cytokines, such as interleukin-6 and tumor necrosis factor-alpha, have been identified in the cerebrospinal fluid and optic nerves of individuals with glaucoma [33]. This indicates that glaucoma may entail a more extensive inflammatory response within the CNS, akin to patterns seen in chronic neurodegenerative disorders. These findings endorse the investigation of anti-inflammatory and metabolic treatments as potential neuroprotective approaches in glaucoma management and further substantiate the case for reclassifying glaucoma as a systemic neurodegenerative condition. Table 1 summarizes research articles evaluating central nervous system involvement in glaucoma using advanced neuroimaging and spectroscopy techniques.

## 7. Common Neurodegenerative Mechanisms

Glaucoma exhibits some pathogenic characteristics akin to those of conventional neurodegenerative disorders, notably Alzheimer’s and Parkinson’s diseases. A notable intersection exists in the accumulation of misfolded proteins. Numerous investigations have shown elevated beta-amyloid levels in the retina and optic nerves of glaucoma patients, akin to the characteristic plaques observed in Alzheimer’s disease [34]. Hyperphosphorylation of tau protein, a characteristic of Alzheimer’s pathology, has also been seen in glaucomatous tissue. The analogies indicate that protein misfolding and aggregation may constitute a shared mechanism of neuronal toxicity in both illnesses, potentially influenced by analogous genetic or environmental variables [35].

Glaucoma and Alzheimer’s disease display analogous patterns of mitochondrial malfunction. Mitochondria are essential for neuronal life by managing energy synthesis, calcium balance, and apoptotic signals. In glaucoma, data from ocular and central tissues demonstrate compromised mitochondrial dynamics, characterized by an altered fission-fusion equilibrium and impaired mitophagy [36]. These alterations reflect those seen in the hippocampus and cortex of Alzheimer’s patients, reinforcing the hypothesis that energy dysregulation is a common catalyst of neurodegeneration. The gradual damage in both illnesses, originating in discrete areas and disseminating through susceptible networks, further corroborates this mechanistic match [37].

Genetic research has commenced to uncover common risk factors linking glaucoma and neurodegenerative disorders. Variants in the APOE gene, especially the ε4 variant correlated with Alzheimer’s disease, have been associated with heightened vulnerability to glaucoma and accelerated progressive rates [38]. Moreover, genes associated with synaptic transmission, inflammation, and oxidative stress regulation have been linked to both illnesses. These findings underscore the possibility of shared genetic predispositions that affect vulnerability to neurodegeneration in both the ocular and cerebral regions. Significantly, they create the potential for targeted medicines that could address both glaucoma and neurological disorders [39].

Ultimately, neurovascular dysfunction constitutes a common element linking glaucoma to more extensive neurodegenerative pathology. Decreased cerebral blood flow and compromised autoregulation have been noted in glaucoma patients, especially within the visual cortex and posterior cerebral circulation [40]. This vascular insufficiency is characteristic of Alzheimer’s disease and may exacerbate hypoxia-induced neuronal stress and inflammatory responses. The interplay of protein aggregation, mitochondrial dysfunction, hereditary predisposition, and vascular impairment indicates that glaucoma and central neurodegenerative disorders are interconnected components of a broader spectrum of systemic neural susceptibility.

Neurovascular variables have become significant moderators of glaucomatous susceptibility. Reduced ocular perfusion pressure (OPP)—determined by mean artery pressure and intraocular pressure—has been linked to a heightened incidence of open-angle glaucoma and accelerated structural and functional progression in several cohorts, but findings differ among populations and techniques. Perfusion-MRI and arterial spin labeling investigations have demonstrated modified cerebral blood flow and neurovascular coupling in primary visual and higher-order cortices in glaucoma, indicating a connection between systemic/central perfusion and disease manifestation [41,42,43].

Large-vessel disease can additionally impair both brain and ocular hemodynamics. Carotid atherosclerosis and stenosis diminish blood flow to the anterior and, through the circle of Willis, posterior circulations, and have been linked to structural retinal alterations and an elevated incidence of open-angle glaucoma in population-based studies. Vertebral artery stenosis, a significant contributor to posterior-circulation ischemia, may reduce perfusion to the visual brain and its associated pathways. Although macrovascular illnesses frequently lead to neuro-ophthalmic abnormalities (e.g., homonymous field defects) that differ from glaucoma, they may serve as systemic stresses that heighten optic nerve vulnerability in predisposed people, particularly those with normal-tension phenotypes. These factors necessitate a meticulous evaluation of cardiovascular risk, blood pressure patterns, and carotid-vertebral patency in individuals exhibiting disease progression despite seemingly sufficient intraocular pressure management [41,42,43].

A growing body of research suggests that perfusion abnormalities can concurrently affect both the eye and the brain. Hypoperfusion of the optic nerve head has been demonstrated to precede the thinning of the retinal nerve fiber layer in preclinical diabetic retinopathy, highlighting the potential causal role of vascular compromise in neurodegeneration [44]. Quantitative OCT angiography offers objective measurements of ocular blood flow, consistently demonstrating diminished flow density in glaucomatous eyes. As reviewed by Brücher et al., these metrics are affected by both systemic and local factors and may indicate cerebrovascular as well as ocular hemodynamics [45]. Studies carried out in 2019 have characterized normal-tension glaucoma as a condition in which an imbalance in brain–eye perfusion and central nervous system susceptibility are pivotal factors [46]. These results collectively emphasize that restricted vascular supply can cause harm from the eye to the brain, underscoring the necessity for comprehensive vascular evaluation in glaucoma research and management.

## 8. Limitations

Although the evidence substantiating glaucoma as a central nervous system condition is expanding, many significant limitations must be recognized. The prevailing research predominantly utilizes cross-sectional study designs, hence limiting the capacity to infer causality. In the absence of longitudinal data, it is uncertain if alterations in the brain occur prior to or subsequent to retinal impairment, or if they develop concurrently as a result of a shared underlying process. This temporal uncertainty hinders the accurate identification of the sequence of pathogenic events in glaucoma and restricts our comprehension of whether central alterations could function as early biomarkers or predictors of disease development.

Secondly, there exists considerable variability in the imaging techniques utilized among research. Variations in scanner strength, voxel resolution, and preprocessing protocols might result in discrepancies in reported outcomes, especially for small structural or functional alterations. Furthermore, certain studies employed limited sample sizes or failed to incorporate adequately matched control groups for age, sex, and vascular risk factors, hence introducing possible bias and diminishing generalizability. The discrepancies in defining glaucoma severity and the utilization of several clinical indicators (such as intraocular pressure, visual field index, and retinal thickness) exacerbate the challenges of cross-study comparisons and meta-analytical synthesis.

Third, although neuroimaging results are insightful, they frequently lack direct pathological connection. Limited investigations have corroborated MRI-detected alterations with histological evidence from human brain tissue, hence constraining confidence in the interpretation of cortical atrophy, metabolic irregularities, or connection disruptions. The clinical significance of the reported changes is not always evident. It remains uncertain if diminished cortical volume in associative visual regions directly causes functional visual impairment or signifies a subsequent, compensating mechanism. The disparity between imaging indicators and clinical symptoms is a hurdle for converting research findings into practical interventions.

The majority of research concentrates on primary open-angle glaucoma, with limited information regarding other subtypes, including normal-tension glaucoma, angle-closure glaucoma, and secondary glaucoma. These forms may vary in their engagement with the central nervous system, and in the absence of comprehensive investigations, generalizing the findings is challenging. Moreover, demographic factors including ethnicity, systemic comorbidities, and genetic predisposition are inadequately investigated concerning central neurodegeneration in glaucoma. Future research must address these limitations to elucidate the scope, processes, and implications of brain involvement in glaucoma and to enhance its classification as a neurodegenerative illness.

Finally, a limited number of the studies included have systematically assessed the prevalence of comorbid neurodegenerative diseases, such as Alzheimer’s or Parkinson’s disease, in glaucoma patients. A recent systematic review reported dementia prevalence between 2.5% and 3.3% in glaucoma populations, with cognitive impairment observed in 12.3% to 90.2% of cases [47]. However, methodological heterogeneity and the absence of standardized cognitive assessments limit the reliability of these estimates. Thus, it is difficult to determine whether the reported brain alterations are exclusively due to glaucoma or partly reflect concomitant neurodegenerative pathology.

## 9. Prospective Outlooks

The acknowledgment of glaucoma as a neurodegenerative disorder encompassing both ocular and central nervous system elements presents numerous intriguing opportunities for future research and clinical use. Longitudinal neuroimaging investigations will be essential to ascertain the temporal link between retinal and brain alterations. Determining whether structural and functional brain changes precede visual field loss or result from it could elucidate glaucoma’s natural history and reveal early biomarkers. Furthermore, these investigations should focus on delineating disease trajectories, identifying early neurodegenerative markers, and assessing therapy responses by quantitative imaging measures.

Improvements in imaging technology will be crucial. Ultra-high-field MRI scanners, such as those functioning at 7 Tesla, provide enhanced spatial resolution that may for more precise evaluation of diminutive brain regions, including the lateral geniculate nucleus and optic radiation. The integration of diffusion imaging, resting-state functional MRI, and spectroscopy in multimodal protocols will enhance comprehension of the interactions among structural deterioration, functional disconnection, and metabolic dysregulation. The amalgamation of these techniques with artificial intelligence and machine learning methodologies may augment the identification of nuanced cerebral alterations and promote personalized disease modeling and prognosis.

From a therapeutic perspective, the neurocentric approach promotes the investigation of neuroprotective and neuroregenerative techniques aimed at both the brain and the eye. Clinical trials are necessary to assess medicines that influence mitochondrial function, protein aggregation, or inflammation—such as nicotinamide, coenzyme Q10, or anti-tau therapies—in glaucoma populations. These methodologies may gain from biomarker-driven patient selection and neuroimaging outcome assessments, thereby advancing precision medicine in glaucoma treatment.

Finally, there is increasing interest in the systemic factors affecting glaucoma etiology, including as vascular dysregulation, sleep disturbances, and metabolic syndromes. Examining the influence of these parameters on cerebral circulation, glymphatic clearance, and neuroinflammation in glaucoma may reveal new modifiable risk factors and inform comprehensive patient care. Collaboration among ophthalmologists, neurologists, radiologists, and cognitive scientists will be crucial to elucidate the intricacies of glaucoma as a neurological disorder. Interdisciplinary initiatives are essential for shifting from a pressure-focused paradigm of glaucoma to a comprehensive neurodegenerative framework that includes ocular, brain, and systemic aspects.

## 10. Conclusions

The dominant perception of glaucoma as an ophthalmic condition focused on lowering intraocular pressure is progressively being contested by an expanding array of information highlighting its neurodegenerative characteristics. Neuroimaging, metabolic, and neuropathological investigations have uncovered structural, functional, and biochemical anomalies that encompass the whole visual pathway, including the lateral geniculate nucleus, visual cortex, and higher-order associative regions. These results align with processes identified in traditional neurodegenerative disorders, including mitochondrial failure, protein misfolding, neuroinflammation, and synaptic disconnection.

Comprehending glaucoma from this perspective has significant clinical ramifications. It underscores the necessity for diagnostic frameworks that encompass not only the retina and optic nerve but also brain imaging and cognitive evaluation. This also paves the way for neuroprotective therapies that extend beyond intraocular pressure regulation to target more extensive neurodegenerative processes. Therapeutic approaches derived from Alzheimer’s or Parkinson’s research may have novel uses in glaucoma, especially for individuals exhibiting progression despite regulated intraocular pressure. Furthermore, biomarkers obtained from brain imaging or cerebrospinal fluid analysis may enhance early detection, illness monitoring, and personalized treatment.

This developing paradigm highlights the significance of interdisciplinary collaboration in glaucoma research and treatment. Incorporating knowledge from neurology, radiology, geriatrics, and ophthalmology is crucial to comprehensively clarify the pathophysiological connections between glaucoma and other neurodegenerative disorders. As our comprehension of the propagation of glaucomatous damage inside brain systems improves, the prospect of earlier and more effective intervention emerges, safeguarding not only vision but also potentially enhancing broader neurological function and quality of life.

In the future, glaucoma classification may improve by integrating pathophysiology-based descriptors with conventional clinical categories. Defining subtypes based on dominant mechanisms—such as intraocular pressure-dependent optic neuropathy, vascular-supply-predominant optic neuropathy, or lamina cribrosa/biomechanics-driven optic neuropathy—can enhance a mechanistic comprehension of disease heterogeneity. While existing criteria maintain established terminology for uniformity and comparability in studies, such mechanistic labels may prove beneficial for study stratification, biomarker development, and personalized management techniques. Traditional terminology and pathophysiological descriptions should be regarded as complementing rather than contradictory, illustrating the multifaceted character of glaucoma.

In conclusion, accumulating evidence suggests that glaucoma may extend beyond an isolated ocular condition and instead involve broader central nervous system processes. While further research is needed to fully elucidate these mechanisms, this evolving perspective has the potential to reshape how we diagnose, monitor, and treat the disease. Glaucoma within a neurodegenerative context may ultimately align it more closely with other complex CNS disorders, encouraging integrated approaches that address both retinal and cerebral components of the disease.

## Figures and Tables

**Table 1:** Summary of key studies investigating central nervous system alterations in glaucoma.

Ref.	Author, Year	N (Patients/Controls)	Methodology	Brain Region(s) Studied	Key Findings
[2]	Haykal et al., 2021	12 POAG/14 controls	Diffusion MRI	Optic tracts, Optic radiations	POAG patients showed progressive fiber density loss.
[4]	Wang et al., 2016	25 POAG/25 controls	T1-weighted MRI	LGN (bilateral), V1 (right), Amygdala (left), Frontal pole cortex	ROI analysis showed volume reduction in LGN, right V1 cortical thinning, left amygdala shrinkage; SBA found reduced cortical thickness in right frontal pole; VBA alone found no GMV differences
[5]	Garaci et al., 2009	16 POAG/10 controls	3-T DTI	Optic nerve, optic radiation	Higher MD and lower FA in optic nerves and radiations in glaucoma
[6]	Engelhorn et al., 2011	50 glaucoma/50 controls	DTI	Optic radiation	44% of glaucoma patients showed significant rarefaction of optic radiation, with volume reduced to 67 ± 16% of controls
[7]	Sidek et al., 2014	30 mild glaucoma/30 severe glaucoma/30 controls	DTI	Optic nerve, optic radiation	Progressive decrease in FA and increase in MD with advancing glaucoma severity; FA in the optic nerve had 87% sensitivity and 80% specificity for detecting severity levels
[8]	Dai et al., 2013	22 POAG/22 controls	rs-fMRI	BA17 (V1), BA18/19, BA7; plus associations with temporal, occipital, motor, and cerebellar regions	BA17 showed reduced connectivity with visual, temporal, sensorimotor, cerebellar areas; increased connectivity with cerebellum, frontal regions; BA18/19 lost connectivity with cerebellar vermis, temporal areas, insula
[15]	Yu et al., 2015	17 severe POAG/20 mild POAG/20 controls	MRI	Visual cortex: V1, V2, V3v, V4, V5/MT+	Mild POAG showed thinning in bilateral V5/MT+; severe POAG showed thinning in left V1, bilateral V2, and V5/MT+; V2 thinner in severe vs. mild
[16]	Boucard et al., 2009	8 POAG/12 controls	MRI, VBM	Visual cortex	GM density reductions in anterior medial occipital cortex matching peripheral field loss
[17]	Frezzotti et al., 2014	13 advanced POAG/12 controls	MRI (VBM, DTI, rs-fMRI)	Visual and extra-visual cortex	WM damage in visual and non-visual tracts, GM atrophy in visual and distant regions, decreased FC in visual, working memory, attention networks, increased FC in visual and executive networks
[18]	Gupta et al., 2009	10 glaucoma/8 controls	1.5T MRI	LGN	LGN height significantly reduced in glaucoma
[20]	Zhou et al., 2017	11 mild–moderate POAG/11 controls	Structural and DTI	Primary visual cortex (V1); optic tract; optic radiation	V1 area and volume reduced in POAG; AD and RD trends not statistically significant
[22]	Yu et al., 2013	36 POAG/40 controls	Structural MRI	Visual cortex	Bilateral cortical thinning in anterior calcarine cortex (left BA17/18, right BA17), smaller areas in left BA37, BA19; correlated with RNFL thickness
[23]	Yang et al., 2024	70 POAG/45 controls	Surface-based dynamic FC (rs-fMRI)	Visual and non-visual cortex	Decreased functional stability in visual network; increased stability in inferior parietal gyrus and right inferior frontal cortex
[24]	Wang et al., 2020	36 glaucoma/20 controls	T1 and rs-fMRI	Lateral, medial, and occipital visual networks	Reduced connectivity between primary visual cortex and higher-order areas
[25]	Giorgio et al., 2018	17 NTG/17 POAG/29 controls	Multimodal MRI: VBM, DTI, rs-fMRI	Visual and non-visual networks	NTG and POAG showed reduced GM volumes and altered connectivity (lower FA/higher diffusivity) in visual and non-visual regions
[26]	Chen et al., 2024	44 PACG/44 controls	rs-fMRI (graph theory)	Whole-brain network	Altered nodal metrics in prefrontal, occipital, temporal, cerebellar regions; intra- and inter-module connectivity changes; no global network metric differences
[28]	Li et al., 2023	34 PACG/34 controls	rs-fMRI (dynamic)	Primary visual cortex (V1) and bilateral calcarine sulcus	Increased dynamic FC between V1 and bilateral calcarine areas
[29]	Aksoy et al., 2019	25 glaucoma/16 suspected glaucoma/16 ocular hypertension/30 controls	MRS	LGN, visual cortex	Lower NAA in LGN in glaucoma and GS; lower Cho in LGN in glaucoma; NAA in VC negatively correlated with cup-to-disk ratio
[30]	Guo et al., 2018	23 early POAG/21 controls	MRS	Visual cortex	Increased Glx/Cr and decreased Ins/Cr ratios in glaucoma; no NAA/Cr or Cho/Cr changes
[31]	Sidek et al., 2016	15 severe glaucoma/15 mild glaucoma/15 controls	MRS	Optic radiation	No significant differences in NAA, Cho, Glx or ratios to Cr

Abbreviations: POAG—primary open-angle glaucoma; LGN—lateral geniculate nucleus; V1—primary visual cortex; ROI—region of interest; SBA—surface-based analysis; VBA—voxel-based analysis; GMV—gray matter volume; 3T—3 Tesla; DTI—diffusion tensor imaging; MD—mean diffusivity; FA—fractional anisotropy; rs-fMRI—resting-state functional magnetic resonance imaging; BA—Brodmann area; V2, V3v, V4, V5/MT+—visual cortical areas; VBM—voxel-based morphometry; GM—gray matter; WM—white matter; FC—functional connectivity; 1.5T—1.5 Tesla; AD—axial diffusivity; RD—radial diffusivity; RNFL—retinal nerve fiber layer; NTG—normal-tension glaucoma; PACG—primary angle-closure glaucoma; FC—functional connectivity; GS—glaucoma suspect; OHT—ocular hypertension; MRS—magnetic resonance spectroscopy; NAA—N-acetyl aspartate; Cho—choline; VC—visual cortex; Glx—glutamate + glutamine; Cr—creatine; Ins—myo-inositol.

## Data Availability

No new data were created or analyzed in this study. Data sharing is not applicable to this article.
